# Efficacy of Multiple Micronutrients Fortified Milk Consumption on Iron Nutritional Status in Moroccan Schoolchildren

**DOI:** 10.1155/2015/690954

**Published:** 2015-08-19

**Authors:** Imane El Menchawy, Asmaa El Hamdouchi, Khalid El Kari, Naima Saeid, Fatima Ezzahra Zahrou, Nada Benajiba, Imane El Harchaoui, Mohamed El Mzibri, Noureddine El Haloui, Hassan Aguenaou

**Affiliations:** Joint Unit of Nutrition and Food Research (URAC39), Ibn Tofaïl University-CNESTEN, Regional Designated Center for Nutrition (AFRA/IAEA), Kenitra, 14000 Rabat, Morocco

## Abstract

Iron deficiency constitutes a major public health problem in Morocco, mainly among women and children. The aim of our paper is to assess the efficacy of consumption of multiple micronutrients (MMN) fortified milk on iron status of Moroccan schoolchildren living in rural region. Children (*N* = 195), aged 7 to 9 y, were recruited from schools and divided into two groups: the nonfortified group (NFG) received daily a nonfortified Ultra-High-Temperature (UHT) milk and the fortified group received (FG) daily UHT milk fortified with multiple micronutrients including iron sulfate. Blood samples were collected at baseline (T0) and after 9 months (T9). Hemoglobin (Hb) was measured *in situ* by Hemocue device; ferritin and C Reactive Protein were assessed in serum using ELISA and nephelometry techniques, respectively. Results were considered significant when the *p* value was <0.05. At T9 FG showed a reduction of iron deficiency from 50.9% to 37.2% (*p* = 0.037). Despite the low prevalence of iron deficiency anemia (1.9%); more than 50% of children in our sample suffered from iron deficiency at baseline. The consumption of fortified milk reduced the prevalence of iron deficiency by 27% in schoolchildren living in high altitude rural region of Morocco. *Clinical Trial Registration.* Our study is registered in the Pan African Clinical Trial Registry with the identification number PACTR201410000896410.

## 1. Introduction

Anemia is recognized as the most common nutritional deficiency worldwide. There are 2 billion people (>30% of the world's population) suffering from anemia [1]. Infants and preschoolers are at major risk, especially in the developing countries. Iron deficiency (ID) is the major cause of anemia and both anemia and iron deficiency in infants and young children are associated with adverse effects on neural development [2]. Inadequate diet due to low iron intake and/or bioavailability is its main etiology [3]. In Morocco, according to a Sentinel Survey for Monitoring and Evaluation of the Fortification Process conducted in 2006–2008, 31.5% of children under 5 y of age suffered from anemia [4]. This prevalence did not improve since the last national survey conducted in 2000 where 31.6% of children aged 6 m–5 y were anemic [5].

In 2001 the Moroccan Ministry of Health had developed and implemented the National Program of Fight against Micronutrients Deficiencies (NPFMD), including iron deficiency, which consisted of iron supplementation of children suffering from anemia, nutritional education, and fortification of staple foods commonly consumed by the entire population.

Supplementation programs and health education to change dietary practices in preschool children have achieved limited success [6]; hence, wheat flour was fortified with elemental electrolytic iron and B group vitamins to improve iron status among Moroccan population [7]. The impact study conducted in 2006–2008 revealed that the fortification of flour with elemental iron did not have a significant effect on the reduction of the prevalence of iron deficiency and iron deficiency anemia in children aged 2–5 y. This was mainly due to several factors, for example, the weak bioavailability of iron used in fortification, Moroccan culinary habits, and the widespread use of flour produced by artisanal mills that are not complying with fortification strategy [8]. It has been therefore recommended to replace the form of iron used for wheat flour fortification by one that is more bioavailable [9].

In 2011, the Ministry of Health launched the National Nutrition Strategy (NNS) 2011–2019 with the aim to improve the nutritional status of the Moroccan population. The objective set for iron deficiency anemia was to reduce its prevalence by 1/3 by 2019 compared to its level in 2011 [10].

However, the accomplishment of the objectives of NNS and NPFMD would not be possible without the participation of other actors in order to target specific vulnerable populations like schoolchildren. Therefore, the Foundation for Child Nutrition (FCN), in partnership with the Ministry of National Education (MNE) and the Ministry of Health (MH), started distributing milk fortified with multiple micronutrients to schoolchildren living in rural regions in Morocco most affected by malnutrition. More than 23.500 children benefited from this intervention.

Hence, in partnership with the FCN, the MNE, and the MH, our team undertook a study to assess the efficacy of the consumption of MMN fortified milk (including iron sulfate) on iron nutritional status of schoolchildren living in rural mountainous regions of Morocco.

## 2. Materials and Methods

### 2.1. Study Design

The study is a longitudinal interventional, placebo-controlled double blind one conducted among schoolchildren (*N* = 195), aged from 7 to 9 years. It lasted for 9 months from February to October 2012. Children were eligible for the study if they were 7 to 9 years old and were not taking supplements during the period of study. Children presenting signs of severe malnutrition or anemia (Hb < 9 mg/dL) were excluded from the study (and transferred to a local health center for follow-up).

The study got the approval of the Ministry of National Education. The purpose and the protocol of the study were presented and explained to the local authorities, regional medical representatives, school Head Masters, teaching staff, and parent's union representatives in schools who in turn explained clearly the benefits of the study to children's parents.

Subsequently, oral and written consents were obtained from children and their parents, respectively, before the beginning of the survey.

Trained medical technicians were recruited from the regional local facilities to help with the samples collection.

### 2.2. Site of the Study

The region where the study took place is situated in the center of Morocco, it is 400 to 700 m above sea level, the climate is continental, and the rainfall varies between 300 and 750 mm per year. Farming is the dominant activity of the population (78.2% of labor force is rural in 2011) [11].

The region is also known to have low-income communities and high prevalence of micronutrient deficiencies [12]. More than one-third of children aged under 5 y suffer from stunting whereas the national prevalence is 23.7% [13].

### 2.3. Selection of Schools

The schoolchildren were recruited from primary schools that were selected on the basis of the following criteria: accessibility to our field team and to the milk distributors, large attendance of schoolchildren enough to cover the required number for the study age range, climatic conditions mainly to avoid interruption of milk distribution by unforeseeable weather, and similarity of socioeconomic and living conditions. Schoolchildren were divided into two groups to receive either the fortified or the nonfortified milk. A distance of 52 km separated the two sites to avoid errors of distribution and/or exchange of milk batches between schoolchildren.

### 2.4. Sample Size

This paper represents one arm of a multiple armed survey aiming to evaluate the nutritional status of schoolchildren with regard to several micronutrients, namely, vitamins A and D, iron, and iodine. Accordingly, the calculation of the sample size was based on the standard deviation (0.6 *μ*mol/L) of the serum level of vitamin A previously determined in a reference regional study on the impact of the consumption of oil fortified with vitamin A on the nutritional status of childbearing women done in 2006–2008 in Morocco. To observe a difference of 0.4 *μ*mol/L with 5% level of significance and 80% power between the intervention and control groups, and after accounting for 20% dropouts, a sample size of 43 children per group was required.

### 2.5. Milk Composition

Two batches of milk were developed and produced for the purpose of the survey. Both fortified and nonfortified milk were identical in appearance, taste, and smell and had the same packages. The only difference was in the nutritional composition as presented in Table 1.

The amount of fortificant added to the fortified milk to obtain the 30% coverage of RDI was determined based on the guidelines of the European Council 2008/100/CE relative to nutritional food labeling [14].

The macro- and micronutrient contents of each batch of milk were doubly checked by Aquanal (Laboratoire Aquitaine Analyses) in France and LOARC (Laboratoire Officiel d'Analyses et de Recherches Chimiques, Casablanca) in Morocco before the beginning and in midsurvey.

### 2.6. Allocation of Groups

195 children were assigned to one of the two groups to receive either fortified milk or nonfortified milk by random drawing of schools.

Children in both Fortified Milk Group (FG) and Nonfortified Milk Group (NFG) received daily 200 mL of whole UHT fortified or nonfortified milk, respectively, during the 9 months of the survey (including weekends and vacation days).

Each child was attributed a code and received daily the corresponding type of milk. The distribution of milk was supervised either by the school principal or the teacher in charge. A separate list was prepared for absent children and their milk was delivered to them at the end of the day. Before weekends and vacation days, a quantity of milk sufficient to cover the period was delivered to the parents of the children.

### 2.7. Data Collection

#### 2.7.1. Socioeconomic Questionnaire (SES)

The data on socioeconomic standards and living conditions of the children and their families were collected at baseline by interviewing the parents. We used an adequate questionnaire that was adapted from other questionnaires used nationally to serve the purpose of our survey. Information collected included the level of education of parents, household size, household monthly global expenses, and alimentary expenses.

#### 2.7.2. Anthropometric Measurements

Anthropometric measurements were taken following standard procedures [15] at baseline. Height was recorded to the nearest 0.1 cm using a stadiometer (Fazzini-2 meters) and weight was recorded to the nearest 0.1 kg using a portable scale (Seca 750-Germany). Stunting and thinness were defined as Height-for-Age (HAZ) and Body Mass Index-for-Age (BAZ) *Z*-scores <–2, respectively, according to the World Health Organization (WHO) [16].

### 2.8. Biochemical Analyses

#### 2.8.1. Hb Measurements

Hb analysis was done at baseline and end line. It was performed* in situ* using the Hemocue portable spectrophotometer (HemoCue AB, Angelholm, Sweden) on a drop of venous blood withdrawn while doing the blood sampling. Anemia was defined as Hb levels <11.5 mg/dL [16]. Hb values measured were adjusted for altitude [17].

#### 2.8.2. Blood Sampling

Whole blood (8 mL) was collected in dry tubes from nonfasting children at baseline and endpoint by venipuncture. Directly after collection, the samples were centrifuged at 5000 rpm for 5 mn and serum was aliquoted in Cryovial tubes and transferred in isothermic box under 4–8°C to the laboratory and then stored at −80°C until analysis of serum Ferritin (SF) and C Reactive Protein (CRP). All the analyses were performed in laboratories of UMRNA (Unité Mixte de Recherche en Nutrition et Alimentation URAC39, Université Ibn Tofail-CNESTEN, Kénitra-Rabat, Morocco).

*(1) Assessment of Serum Ferritin*. Quantitative determination of ferritin level in the serum was performed in the laboratory using a colorimetric immunoenzymatic method type ELISA sandwich (NovaTec Immundiagnostica GMBH, Germany). Iron deficiency was defined as serum ferritin <15 *μ*g/L and iron deficiency anemia was defined as iron deficiency along with anemia [16].

*(2) Serum Concentrations of CRP*. The level of CRP in the serum was determined by nephelometry using the Minineph kit (MININEPH, Références, ZK044.L.R, The Binding Site, Birmingham, UK). In our survey CRP serves mainly as a biomarker of inflammation or subclinical infection on days of blood sampling. A cutoff of >10 mg/L was used for abnormal serum CRP concentrations [18].

### 2.9. Statistical Analysis

Data analysis was done by the software IBM SPSS Statistics version 20 (Statistical Package for the Social Sciences). Anthropometric measurements were analyzed by Anthro+ (WHO standards) [16]. The distribution normality of the quantitative variables was tested by Kolmogorov-Smirnov test. The variables normally distributed were presented as mean ± standard deviation and those nonnormally distributed as median (interquartile range). ANOVA was used to compare variances between independent samples. The homogeneity of variances was tested using Leven's test, and the correction of Welch was used in the case of nonhomogeneous variances. Mann-Whitney test was used to compare independent samples for variables nonnormally distributed. Wilcoxon test was used to compare the relation between T0 and T9 within the same group. The nominal variables were presented as proportion and 95% Confidence Interval (Lower-Upper). Chi-square test was used to test independence between nominal variables. Chi-square value was corrected for cells with a theoretical frequency less than 5; if a theoretical *n* < 5 we take the *p* value of Fisher, and 95% Confidence Intervals were determined using the Bootstrap technique based on 1000 bootstrap samples. The correlation between high CRP values (>10 mg/L) and high ferritin level was tested using Bivariate Correlations test. A difference was considered as statistically significant if *p* < 0.05.

## 3. Results

**Figure 1:**
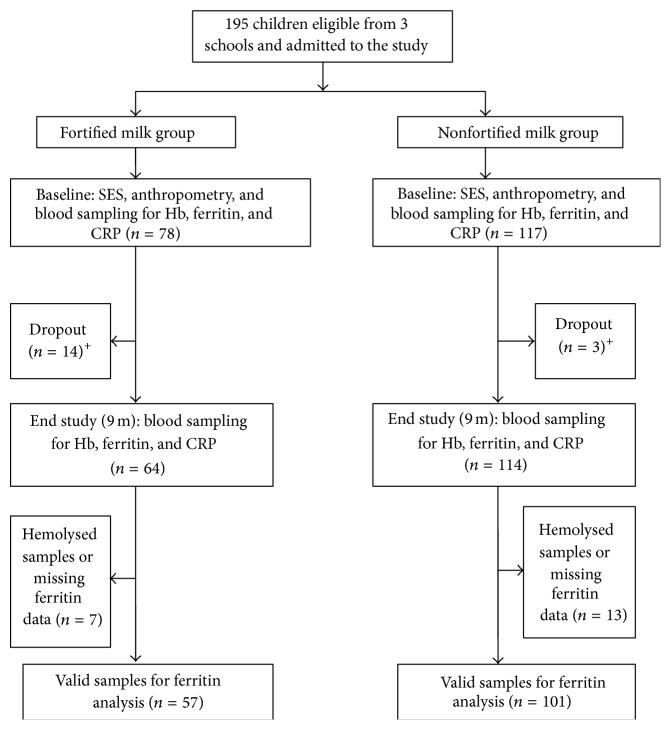
Participant Flowchart: ^+^dropouts: participants who either refused to give blood or were absent on the day of samples withdrawal, changed school or were relocated during study, or were excluded due to severe anemia (Hb < 9 mg/dL). SES: Socioeconomic Status, Hb: Hemoglobin, and CRP: C Reactive Protein.

Figure 1 represents the participant flowchart. The rate of compliance was not the same in both groups; we observed a larger number of dropouts in the fortified group that was due to participants' refusal to continue the survey (children refused to give blood samples or were absent on the day of blood withdrawal), change of school, or relocation out of the study area. Nevertheless, the size of the sample in FG was still statistically valid. No adverse events because of the intervention were reported during the course of the study.

The growth parameters and socioeconomic characteristics are presented in Table 2.

There were no significant differences in baseline anthropometric measurements or socioeconomic characteristics of children between the NFG and the FG.

In general, the prevalence of illiteracy in mothers for both groups was high compared to fathers with 95.2% and 60.7%, respectively.

91.1% of households spend less than 195 US$/m for food compared to 54.4% for general expenditure. 195 US$ is the equivalent of the guaranteed minimum wage for governmental employees.

To assess the dietary habits of our population, we used a food frequency questionnaire that was filled by the children's mothers at baseline. The preliminary analysis of the data collected showed that the majority of children consumed foods rich in iron or that stimulate its absorption (e.g., meat, legumes, and fruits). Dairy products consumption was moderate for yogurt and low for cheese. On the other hand, more than 90% of children consumed tea at least once per week, which could be the reason behind the high prevalence of ID among children. Both groups had similar food trends and the difference in dietary behaviors between FG and NFG was not statistically significant (*p* > 0.05).

### 3.1. Iron Deficiency (Tables 3 and 4)

At T9 FG showed a reduction of the prevalence of iron deficiency (serum ferritin < 15 *μ*g/L) in comparison with T0 from 50.9% (95% CI: 38.6–63.2) to 37.2% (95% CI: 23.3–51.2), while for the NFG it remained stable between T0 and T9 at 56% and 56.4%, respectively. The difference between FG and NFG was statistically significant at T9** (** *p* = 0.035). The *p* value calculated within the same groups between T0 and T9 was significant for the FG** (** *p* = 0.037) and nonsignificant for the NFG (*p* = 0.927).

The median (interquartile) of serum ferritin increased in the FG from 14.0 (9.0; 20.0) at T0 to 17.0 (11.0; 26.0) at T9, while it remained stable in NFG. The difference between the two groups was statistically significant at T9 (*p* = 0.019).

### 3.2. Anemia and Iron Deficiency Anemia (Tables 3 and 4)

The prevalence of iron deficiency anemia (IDA) (Hb < 11.5 mg/dL and ferritin < 15 *μ*g/L) for the FG dropped from 1.8% (95% CI: 0.0–5.3) at T0 to 0.0% at T9. For the NFG, it remained unchanged at 2.0% both at baseline and end line. The difference between both groups was not statistically significant at T9 (*p* = 0.064).

The mean Hb increased slightly in both groups. At T0 it was 14.58 ± 1.58 and 14.22 ± 1.21 for NFG and FG, respectively, whereas at T9 it became 15.05 ± 1.43 and 14.59 ± 1.16.

The difference between the two groups at T9 was not statistically significant (*p* = 0.213).

## 4. Discussion

Our results showed that the consumption of milk fortified with ferrous sulfate and other micronutrients is efficacious in reducing the prevalence of iron deficiency and improving iron status indicators in a sample of children 7–9 y of age. Authors from different countries previously published results of efficacy interventions using fortified milk and reported varying degrees of success in reducing the iron deficiency depending on the dose and duration of intervention.

In Chile, two studies conducted in infants confirmed the efficacy of iron-fortified milk with ferrous sulfate combined with ascorbic acid [19, 20]. While in India, a trial conducted among children aged 1–4 years for a period of one year demonstrated the efficacy of a multiple micronutrients (including iron and zinc) fortified milk on growth, body iron stores, and anemia [21].

In 2003, a study done in Morocco to assess the effect of a dual-fortified salt (DFS) containing iodine and microencapsulated iron on nutritional status of schoolchildren showed that the prevalence of IDA in the fortified group decreased from 35% at baseline to 8% after 40 weeks of intervention (*p* < 0.001) [22].

While two other surveys conducted in Brazil and Sweden revealed a lesser efficacy of fortified milk. In the first one 185 Brazilian children with mild or severe anemia received milk fortified with 3 mg/L of iron amino acid chelate. After 222 days of intervention, 43% remained anemic. The reduced efficacy in this study was attributed to the low level of iron fortification (3 mg/L) [23], while in Sweden, a controlled trial in 36 children treated for 6 months with fortified milk with 7.0 or 14.9 mg/L of iron reported no significant effects on hematological and iron status indicators and this has been explained by the fact that these children had a good baseline iron status; thus, noticeable changes in Hemoglobin or iron status should not be expected [24].

In our trial, we observed an increase of median ferritin levels and a marked reduction in the prevalence of iron deficiency (27%) in FG, compared to NFG, and this has been reported by other fortification trials conducted in low-income countries [25, 26] which highlights a specific effect attributable to the intervention. Also availability of vitamin A, essential for erythropoiesis, could have resulted in a better overall improvement of iron status [27].

However, it is worthy to emphasize that our milk contained naturally a high calcium level (240 mg of calcium per 200 mL) and it is well known that calcium in milk interferes significantly with the absorption of iron. The mechanism of action for absorption inhibition is unknown. Recent analyses of the dose-effect relationship show that the first 40 mg of calcium in a meal does not inhibit absorption of haem and nonhaem iron. Above this level of calcium intake, a sigmoid relationship develops, and at levels of 300–600 mg calcium, it reaches a 60% maximal inhibition of iron absorption [28]. Thus, the effects on the prevalence of anemia and iron status herein described were most probably modulated by the interaction of both iron and calcium at the mucosal cell (at the intestinal level), resulting in a less pronounced efficacy in improving iron than fortification without a high level of calcium. The efficacy could have also been more evident if enhancers of iron absorption had been added to this milk. Ferrous sulfate along with vitamin C, to potentiate bioavailability of iron, added to milk proved to be more effective in reducing the prevalence of anemia in other studies [21, 29, 30].

## 5. Conclusions

This study provides evidence that delivery of iron via a food-based vehicle, milk in this instance, is a feasible option and produces a positive effect on iron status among schoolchildren. It provides a potential strategy for achieving Millennium Development Goals targeting reduction in mortality, morbidity, and malnutrition among children, constituting an example of how the use of research can directly benefit the design of successful public nutrition programs such as the National Nutrition Strategy, the National Program of Fight against Micronutrient Deficiencies, and the application of the recommendations of the second International Conference on Nutrition (ICN2). Indeed, our work may represent a solution at the national level, encouraging the generalized distribution of fortified breakfasts sponsored by the MNE in rural schools. There are indications (according to FCN) that such distribution may result in a reduction of school dropout rates too.

## Limitations of the Study

Because of* a priori* criteria of selection of the schools, we were unable to recruit an equal number of children in both groups (as shown in Figure 1). In spite of this and the small size of the study population our findings were statistically valid. Nevertheless, future studies should try to overcome these limitations.

## Figures and Tables

**Table 1:** Composition of nonfortified and fortified milk.

Nutritional composition	Nonfortified milk		Fortified milk	
	Amount/200 mL serving	% RDI^a^ children 7–9 y	Amount/200 mL serving	% RDI^a^ children 7–9 y
Energy (Kcal)	154.8	—	154.8	—
Fat (%)	5.8	—	5.8	—
Protein (g)	5.8	—	5.8	—
Lipids (g)	6	—	6	—
Carbohydrates (g)	19.44	—	19.44	—
Calcium (mg)	240	30	240	30
Iron (mg)	<0.4	<3	4.2	30
Iodine (*μ*g)	20.8	<14	45	30
Vitamin D3 (*µ*g)	<1	<10	3	30
Vitamin A (*μ*g)	54	<7	240	30

RDI: recommended dietary intake.

^a^The values were based on the guidelines of the European Council 2008/100/CE relative to nutritional food labeling.

**Table 2:** Baseline demographic, anthropometric, and socioeconomic characteristics of schoolchildren enrolled in the study.

	Total (*N* = 195)		NFG (*N* = 117)		FG (*N* = 78)		*p* value
General characteristics							
Age (y) (mean ± SD)	8.0 ± 0.7		8.0 ± 0.7		7.9 ± 0.8		0.371
Baseline anthropometry							
Height (cm) (mean ± SD)	122.3 ± 6.1		121.9 ± 6.3		122.8 ± 5.6		0.352
Weight (kg) (mean ± SD)	23.2 ± 3.0		23.1 ± 3.0		23.2 ± 2.9		0.483
BMI (kg/m^2^) (mean ± SD)	15.4 ± 1.1		15.5 ± 1.0		15.4 ± 1.2		0.358
Nutritional status							
Stunting^a^ HAZ <−2 SD (%)	8.4		6.8		10.3		0.219
Thinness^a^ BAZ <−2 SD (%)	2.1		0		5.1		

	%	95% CI	%	95% CI	%	95% CI	*p* value

Sex							
Female	50.6	(42.9–58.3)	52.4	(42.7–61.2)	47.7	(35.4–60.0)	0.815
Male	49.4	(41.7–57.1)	47.6	(38.8–57.3)	52.3	(40.0–64.6)	
Level of education							
Mother							
Illiterate	95.2	(91.7–98.2)	98.1	(95.1–100.0)	90.8	(83.1–96.9)	0.069
Primary	3.6	(1.2–6.5)	1.0	(0.0–2.9)	7.7	(1.5–15.4)	
Secondary	1.2	(0.0–3.6)	1.0	(0.0–2.9)	1.5	(0.0–4.6)	
Father							
Illiterate	60.7	(53.6–68.5)	60.2	(51.5–68.9)	61.5	(49.2–73.8)	0.562
Primary	31.5	(24.4–38.7)	32.0	(23.3–40.8)	30.8	(18.5–43.1)	
Secondary	7.1	(3.6–11.3)	7.8	(2.9–13.6)	6.2	(1.5–12.3)	
College	0.6	(0.0–1.8)	0.0		1.5	(0.0–4.6)	
Household size							
<6 persons	48.8	(41.1–56.5)	49.5	(39.8–59.2)	47.7	(35.4–60.0)	0.944
6 to 10 persons	51.2	(43.5–58.9)	50.5	(40.8–60.2)	52.3	(40.0–64.6)	
Total monthly expense							
<122US$	25.6	(19.6–32.7)	28.2	(19.4–37.8)	21.5	(12.3–32.3)	0.117
122–195US$	28.6	(21.4–35.7)	21.4	(14.6–29.1)	40.0	(27.7–52.3)	
196–244US$	26.2	(20.2–32.7)	31.1	(22.3–39.8)	18.5	(9.2–29.2)	
245–366US$	11.9	(7.1–16.7)	11.7	(5.8–18.4)	12.3	(4.6–20.0)	
>367US$	7.7	(4.2–11.9)	7.8	(2.9–13.6)	7.7	(1.5–13.8)	
Monthly expense for food							
<110US$	63.1	(55.4–70.2)	60.2	(50.5–69.9)	67.7	(55.4–78.5)	0.076
110–147US$	18.5	(12.5–24.4)	23.3	(15.5–31.1)	10.8	(3.1–18.5)	
148–195US$	9.5	(5.4–14.3)	6.8	(2.9–12.6)	13.8	(6.2–23.0)	
196–305US$	8.3	(4.8–12.5)	9.7	(3.9–16.5)	6.2	(1.5–12.3)	
>306US$	0.6	(0.0–1.8)	0.0		1.5	(0.0–4.6)	

BMI: Body Mass Index; ^a^HAZ and BAZ were calculated by *Anthropo*+.

**Table 3:** Iron status at baseline and endpoint in the study groups.

Biochemical parameters	Total		NFG		FG		*p* value^*∗*^
	*N*	Mean ± SD	*N*	Mean ± SD	*N*	Mean ± SD	
Hemoglobin (mg/dL)							
Baseline	178	14.45 ± 1.46	114	14.58 ± 1.58	64	14.22 ± 1.21	0.090
End line	178	14.88 ± 1.35	114	15.05 ± 1.43	64	14.59 ± 1.16	0.213

	*N*	Median; interquartile	*N*	Median; interquartile	*N*	Median; interquartile	*p* value^*∗*^

Serum ferritin (*μ*g/L)							
Baseline	158	13.0 (9.0; 21.0)	101	13.0 (8.0; 21.0)	57	14.0 (9.0; 20.0)	0.610
End line	144	14.0 (9.0; 22.7)	101	13.0 (8.0; 20.0)	43	17.0 (11.0; 26.0)	0.019

^*∗*^ *p* value by one way ANOVA for means and Mann-Whitney test for medians.

**Table 4:** Prevalence of anemia, iron deficiency anemia, and iron deficiency at baseline and endpoint in both groups.

	Total		NFG		FG		
	%	95% CI	%	95% CI	%	95% CI	*p* value^**∗**^
	*N* = 195		*N* = 117		*N* = 78		
Anemia^a^ (Hb < 11.5 mg/dL)							
Baseline	2.2	(0.6–4.5)	2.6	(0.0–6.1)	1.6	(0.0–4.7)	0.999
End line	2.2	(0.6–4.5)	2.6	(0.0–6.1)	1.6	(0.0–4.7)	0.999
Iron deficiency anemia^b^ (Hb < 11.5 mg/dL and fe < 15 *μ*g/L)							
Baseline	1.9	(0.0–4.5)	2.0	(0.0–5.0)	1.8	(0.0–5.3)	0.760
End line	1.4	(0.0–3.5)	2.0	(0.0–5.0)	0.0	(0.0–0.0)	0.064
Iron deficiency^c^ (Fe < 15 *μ*g/L)							
Baseline	*N* = 158		*N* = 101		*N* = 57		
	54.1	(45.9–62.4)	56.0	(47.0–65.0)	50.9	(38.6–63.2)	0.536
End line	*N* = 144		*N* = 101		*N* = 43		
	50.7	(43.1–59.0)	56.4	(45.6–66.3)	37.2	(23.3–51.2)	0.035
*p* value^**∗∗**^ for iron deficiency within same group			0.927		0.037		

^*∗*^ *p* value for comparing deficiency prevalence among study groups using *χ* ^2^-test.

^*∗∗*^ *p* value for comparing deficiency prevalence within same study group using Wilcoxon test.

^a^Anemia was defined as Hb levels <11.5 mg/dL. ^b^Iron deficiency anemia was defined as iron deficiency along with anemia by the above-mentioned criteria. ^c^Iron deficiency was defined as serum ferritin <15 *μ*g/L.

## Abbreviations

BAZ: Body Mass Index-for-Age *Z*-scores
CRP: C Reactive Protein
DFS: Dual-fortified salt
FCN: Foundation for Child Nutrition
FG: Fortified Milk Group
HAZ: Height for age *Z*-scores
ID: Iron deficiency
IDA: Iron deficiency anemia
MH: Ministry of Health
MMN: Multiple micronutrients
MNE: Ministry of National Education
NFG: Nonfortified Milk Group
NNS: National Nutrition Strategy
NPFMD: National Program of Fight against Micronutrients Deficiencies
RDI: Recommended dietary intake
SD: Standard deviation
SES: Socioeconomic Status
SF: Serum ferritin
SPSS: Statistical Package for the Social Sciences
UHT: Ultra-High-Temperature
WAZ: Weight for age *Z*-scores.
